# The Characteristics of Spike Glycoprotein Gene of Swine Acute Diarrhea Syndrome Coronavirus Strain CH/FJWT/2018 Isolated in China

**DOI:** 10.3389/fvets.2021.687079

**Published:** 2021-07-22

**Authors:** Yan-Yan Guo, Pei-Hua Wang, Yuan-Qing Pan, Rui-Zhu Shi, Ya-Qian Li, Fan Guo, Li Xing

**Affiliations:** ^1^Institutes of Biomedical Sciences, Shanxi University, Taiyuan, China; ^2^Shanxi Provincial Key Laboratory of Medical Molecular Cell Biology, Shanxi University, Taiyuan, China; ^3^Shanxi Provincial Key Laboratory for Prevention and Treatment of Major Infectious Diseases, Taiyuan, China

**Keywords:** phylogenetic analysis, epitope, spike glycoprotein, virus genome, swine acute diarrhea syndrome coronavirus (SADS-CoV)

## Abstract

Swine acute diarrhea syndrome (SADS) is a highly contagious infectious disease characterized by acute vomiting and watery diarrhea in neonatal piglets. The causative agent for SADS is the swine acute diarrhea syndrome coronavirus (SADS-CoV), an alphacoronavirus in the family *Coronaviridae*. Currently, SADS-CoV was identified only in Guangdong and Fujian provinces of China, not in any other regions or countries in the world. To explore the genetic diversity of SADS-CoV isolates, herein we comparatively analyzed 44 full-length genomes of viruses isolated in Guangdong and Fujian provinces during 2017–2019. The spike glycoprotein gene of SADS-CoV strain CH/FJWT/2018 isolated in Fujian province is distinct from that of other viral isolates in either spike glycoprotein gene-based phylogenetic analysis or whole genome-based gene similarity analysis. Moreover, at least 7 predicted linear B cell epitopes in the spike glycoprotein of CH/FJWT/2018 would be affected by amino acid variations when compared with a representative virus isolated in Guangdong province. The spike glycoprotein of coronaviruses determines viral host range and tissue tropism during virus infection via specific interactions with the cellular receptor and also plays critical roles in eliciting the production of neutralizing antibodies. Since SADS-CoVs have a broad cell tropism, the results in this report further emphasize that the spike glycoprotein gene is a pivotal target in the surveillance of SADS-CoV.

## Introduction

The swine acute diarrhea syndrome (SADS) was first reported in Guangdong province, China in 2017 (1–3). It is a highly contagious infectious disease that is characterized by acute vomiting and watery diarrhea in neonatal piglets, which leads to high mortality and significant economic losses for the local pork industry. The causative agent for SADS is the swine acute diarrhea syndrome coronavirus (SADS-CoV), which is also named swine enteric alphacoronavirus (SeACoV) (1) or porcine enteric alphacoronavirus (PEAV) (2). The genome of SADS-CoV is closely related to that of the species *Rhinolophus* bat coronavirus HKU2, both of which are members of the genus *Alphacoronavirus* within the subfamily *Orthocoronavirinae* of family *Coronaviridae* of suborder *Cornidovirineae* of order *Nidovirales. Rhinolophus* bat coronavirus HKU2 was first reported from Guangdong province and Hong Kong in 2004 and 2006, respectively, in China (1, 4). The full-length genome of SADS-CoV is about 27 kb in length with a 5′ cap and a 3′ polyadenylated tail, and contains a 5′ untranslated region (UTR) followed by nine open reading frames (ORFs) and a 3′ UTR (1). ORF1a and ORF1b encode polyprotein 1a (pp1a) and polyprotein 1b (pp1b), respectively, and occupy 5′ two-thirds of the genome. During translation, pp1a and pp1b will be processed into 16 non-structural proteins (Nsp1-16) responsible for the viral RNA synthesis (1, 3). The remaining part of virus genome contains ORFs that encode four structural proteins including spike (S) glycoprotein, envelope (E) protein, membrane (M) protein, and nucleocapsid (N) protein, one bigger accessory protein NS3 and two smaller accessory proteins (NS7a and NS7b) (3, 5). The M and E proteins of coronaviruses are required for the viral particle assembly (6–8). The N protein plays a critical role in the packaging of viral RNA genomes into virus particles via interactions with each other and the viral genomic RNA (9).

The S glycoprotein of coronaviruses is responsible for the entry of virus into host cells by interacting with the cellular receptor. This protein can also elicit the production of neutralizing antibodies in the natural host (10, 11). Thus, the S glycoprotein serves as a primary target in the development of effective vaccines against coronaviruses and a major determinator for the efficacy of vaccines. The monomer S glycoprotein will be cleaved into S1 and S2 subunits during virus entry into host cells. S1 subunit is responsible for cell attachment and receptor binding, whereas S2 subunit mediates the membrane fusion between virus and cell host (10, 11). Thus far, the cellular receptor for S glycoprotein of SADS-CoV is still not identified. The cryo-EM structures of S glycoprotein trimers have been determined for both SADS-CoV and HKU2 (12). The overall structure of S glycoprotein trimer adopts a mushroom-like shape (~150 Å in height and ~115 Å in width), in which the β-sheets of the S1 subunit form a cap, the α-helices of the S2 subunit form a central stalk, and twisted β-sheets and loops of the S2 subunit form a root (12). The S1 subunit of monomer S glycoprotein can be further divided into several subdomains including NTD (N-terminal domain), CTD (C-terminal domain), SD1, and SD2 (12).

The first remarkable outbreak of SADS occurred in newborn piglets in February 2017 in commercial farms around the foot of a mountain located in Guangdong province of southern China, which killed 24,693 piglets (1–3). A retrospective investigation of SADS-CoV infection in pig farms showed that SADS-CoV had emerged in Guangdong province as early as August 2016 (3). In February 2019, SADS-CoV infection re-emerged in pig herds in Guangdong, in which about 2000 pigs were killed at other pig farm near the original farms of the first outbreak (13). In 2018, a new SADS-CoV strain (CH/FJWT/2018) was identified in pig farms in Fujian province neighboring to the Guangdong province (14). In this study, we sought to explore the genomic characteristics in order to understand the genetic relatedness and genomic diversity of SADS-CoV field isolates in China.

## Materials and Methods

### Dataset, Phylogenetic Tree Construction, and Genomic Similarity Analysis

A total of 44 full-length genomic sequences of SADS-CoVs that were isolated in China from 2017 to 2019 were retrieved from GenBank. The sequences were aligned with Clustal Omega (https://www.ebi.ac.uk/Tools/msa/clustalo/) (15). The phylogenetic trees were constructed using the neighbor-joining method in the MEGA-X software (16, 17) based on the whole genomic sequences or the full-length sequences of ORFs encoding pp1a and pp1b, or S, E, M, or N proteins. The viruses in this report were identified by their GenBank accession number, viral name, country, and year of collection (in a format GenBank accession number-virus name/country-year of collection). The phylogenetic inference was tested with the bootstrap method with 1,000 replications. Bootstrap values >50% were indicated. The genomic similarity plot was generated by using Simplot ver. 3.5.1 (18) to compare the whole genomes of SADS-CoV isolates.

### Prediction of Linear B Cell Epitopes

The linear B cell epitopes in the S glycoprotein of SADS-CoV were predicted using BepiPred-2.0 server, which predicts B-cell epitopes using a Random Forest algorithm trained on epitopes and non-epitope amino acids determined from crystal structures (19). The residues with scores above the threshold value (set at 0.5) were predicted to be part of an epitope and shown in yellow on the graph (Y-axes depicts residue scores and X-axes indicates amino acid residue positions in the sequence). Only conserved fragments of more than 5 amino acid residues that were predicted as potential epitopes by BepiPred-2.0 were taken into consideration in this study.

### Three-Dimensional Structure of Partial S Glycoprotein of SADS-CoV

Tertiary structure modeling of part of SADS-CoV S glycoprotein containing S1 subunit and the N-terminus of S2 subunit (aa 1-692 relative to the S glycoprotein of CH/FJWT/2018) was carried out using I-TASER (Iterative Threading ASSEmbly Refinement) server (20–22). I-TASER is a hierarchical approach to protein structure prediction and structure-based function annotation, which first identifies structural templates from the RCSB protein data bank (PDB) by multiple threading approach LOMETS, with full-length atomic models constructed by iterative template-based fragment assembly simulations.

## Results

### Comparative Analysis of Genomes of SADS-CoVs Isolated in China

**Figure 1:**
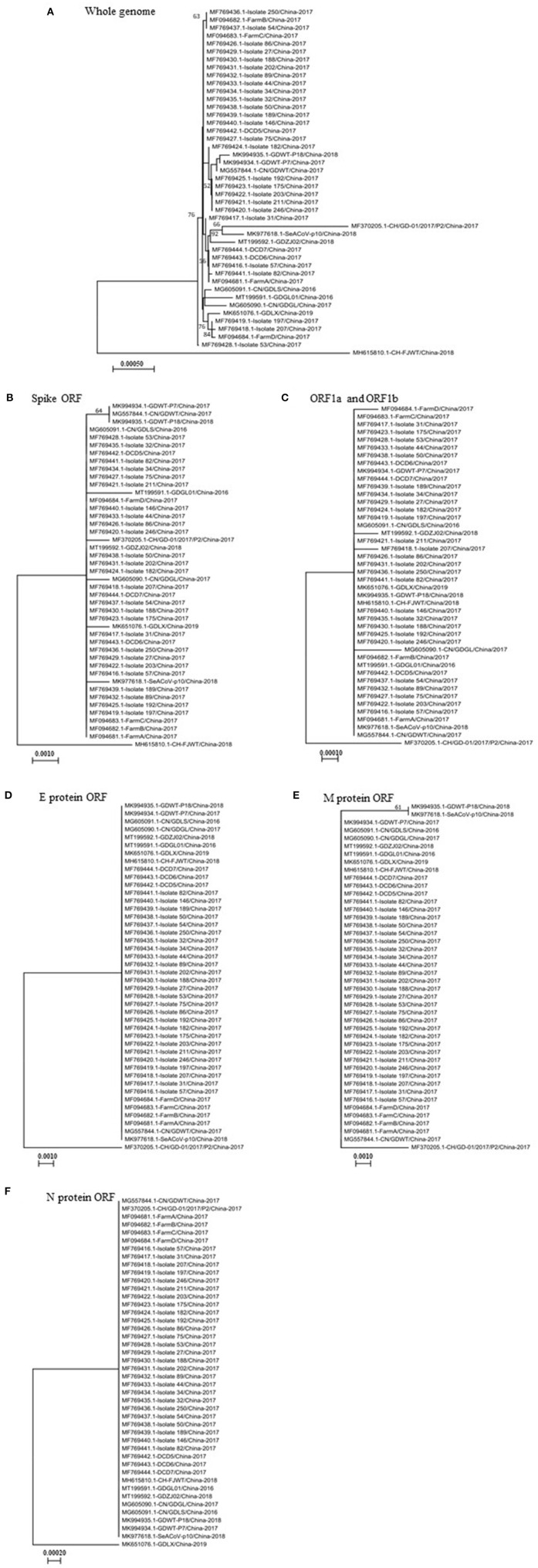
The phylogenetic trees based on the whole genomes of SADS-CoVs **(A)** or the full-length spike glycoprotein ORF **(B)**, ORF1a and ORF1b **(C)**, or E protein ORF **(D)**, M protein ORF **(E)**, or N protein ORF **(F)**. Multiple-sequence alignments were performed using Clustal Omega server and then phylogenetic trees were constructed using neighbor-joining method in the MEGA-X software (16, 17). The numbers at each branch represent bootstrap values >50% of 1,000 replicates. Scale bars indicate the number of inferred substitutions per site. The tree is drawn to scale, with branch lengths in the same units as those of the evolutionary distances used to infer the phylogenetic tree. Evolutionary distances were computed using the Maximum Composite Likelihood method (17).

Thus far, the SADS-CoV was identified only in Guangdong and Fujian provinces in China, not in any other region or countries in the world. The identified sequences in pigs are closely related to the bat HKU 2-like coronavirus sub-lineage (5). To comparatively analyze genetic characteristics of SADS-CoVs, we performed a phylogenetic analysis based on the full-length genome sequence of viral strains isolated in China. The phylogenetic analysis was also done based on the complete sequence of individual ORFs encoding pp1a and pp1b, S, E, M, or N proteins. Results show that the virus CH/FJWT/2018 isolated in Fujian province (GenBank accession number MH615810.1) represents a lineage distinct from other viruses isolated in China in both whole genome-based (Figure 1A) and complete S glycoprotein ORF-based (Figure 1B) phylogenetic trees. However, CH/FJWT/2018 virus belongs to the same genogroup as most of viruses isolated in Guangdong province in the phylogenetic trees based on the complete ORFs encoding pp1a and pp1b (Figure 1C), E (Figure 1D), M (Figure 1E), or N (Figure 1F) proteins.

**Figure 2:**
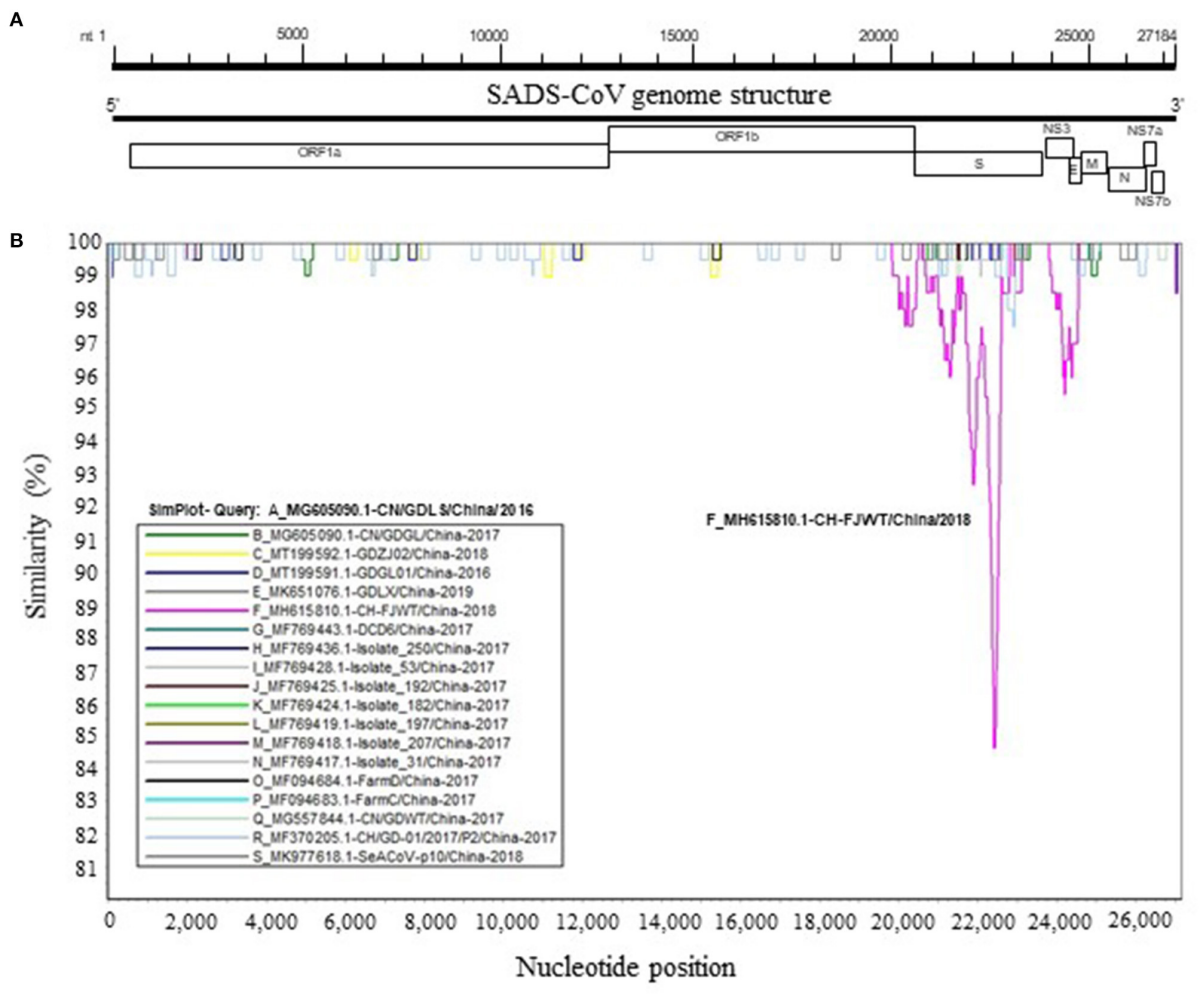
Similarity plot of the full-length genomic sequences of non-identical SADS-CoV isolates. **(A)** Schematic diagram of the genomic structure of SADS-CoV. S, spike glycoprotein ORF; NS3, accessory protein NS3 ORF; E, envelope protein ORF; M, membrane protein ORF; N, nucleoprotein ORF; NS7a, accessory protein NS7a ORF; NS7b, accessory protein NS7b ORF. Numbers indicate the nucleotide position (nt) relative to the genomic sequence of SADS-CoV CH/FJWT/2018. **(B)** Similarity plot of viral genomes was constructed using the two-parameter (Kimura) distance model (18) with a sliding window of 200 bp and step size of 20 bp. Vertical and horizontal axes indicate the percent nucleotide similarity (%) and nucleotide position (nt) in the graph, respectively.

To further analyze genetic characteristics of SADS-CoV strains, the genomic similarity plot was generated using Simplot ver 3.5.1 (18) for all the non-identical full-length genomic sequences. As shown in Figure 2, the S glycoprotein ORF of CH/FJWT/2018 at nt 20,000–25,000 of viral genome shows the lowest similarity between all the viruses included. In addition, the second lowest similarity was found for the NS3 ORF of CH/FJWT/2018. The results demonstrate that S glycoprotein ORF may distinguish CH/FJWT/2018 from all other viruses isolated in China.

### Linear B Cell Epitopes in S Glycoprotein

**Figure 3:**
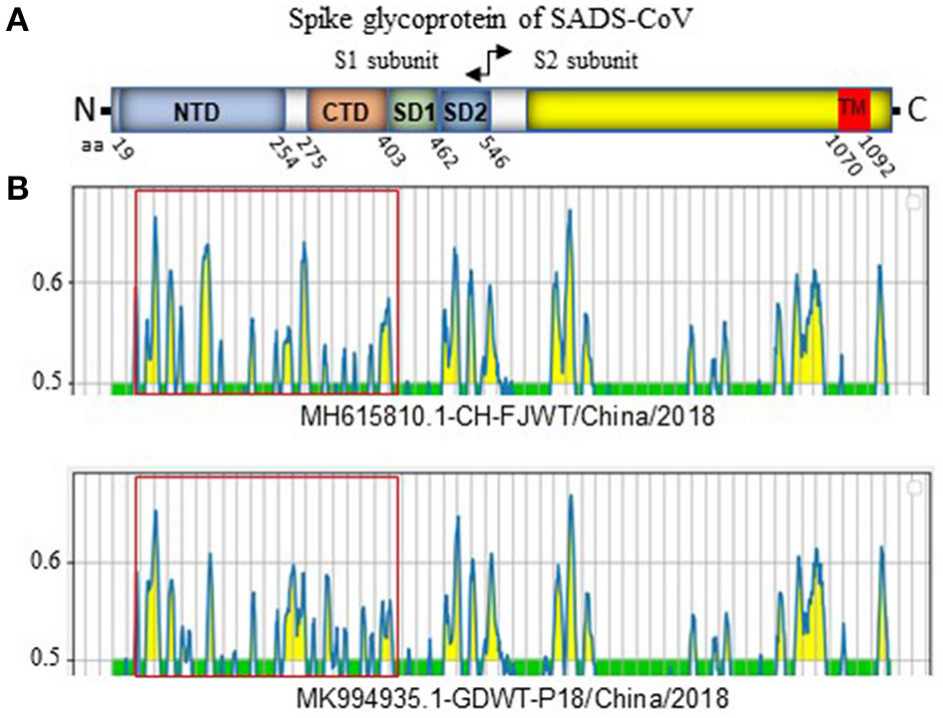
Linear B cell epitope map of full-length S glycoprotein of indicated SADS-CoVs. **(A)** Diagram depicting the main features of SADS-CoV S glycoprotein, which include putative cleavage site between S1 and S2 subunits at aa 546, N-terminal domain (NTD) (aa 19-254), C-terminal domain (CTD) (aa 275-402), SD1 (aa 403-461), SD2 (aa 462-546), and the transmembrane domain (TM, aa 1070-1092). Numbers indicate the amino acid position relative to S glycoprotein of CH/FJWT/2018 strain (GenBank accession number: MH615810.1). **(B)** The linear B cell epitope map was generated by using BepiPred-2.0 server (19). Amino acid residues with scores above the threshold value that was set at 0.5 were predicted to be part of an epitope and colored in yellow on the graph. Y-axes depicts residue scores. X-axes depicts the amino acid positions, which is also relative to the diagram on the top **(A)**.

**Figure 4:**
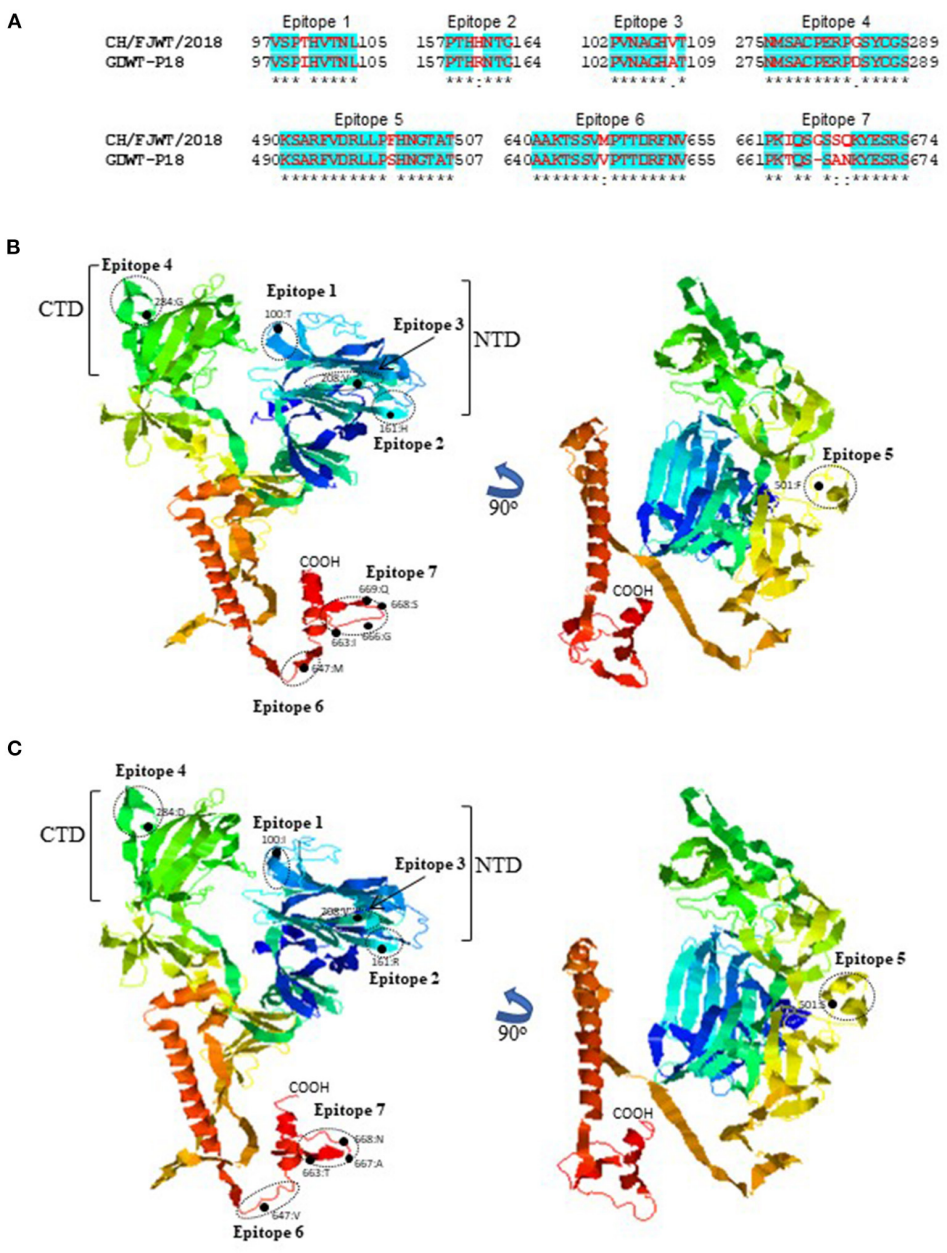
Linear B cell epitopes affected by amino acid variations in the S glycoproteins of SADS-CoVs CH/FJWT/2018 and GDWT-P18. **(A)** The amino acid sequence of predicted linear B cell epitopes are shown with red bold letters. Numbers indicate the amino acid positions relative to the full-length S glycoprotein of indicated SADS-CoVs. 3D view of epitopes in the S1 subunit and the N-terminus of S2 subunit of CH/FJWT/2018 **(B)** or GDWT-P18 **(C)**. 3D structure of S glycoprotein was modeled by using I-TASER server (20–22). The predicted epitopes are circled and the amino acid variations are labeled with black dot. NTD, N-terminal domain (aa 19-254); CTD, C-terminal domain (aa 275-402).

SADS-CoV GDWT-P18 strain (GenBank accession number: MK994935.1) was obtained by propagating in Vero cells a virulent virus CN/GDWT/2017 (GenBank accession number: MG557844) isolated in Guangdong province (23). To analyze potential antigenicity of S glycoprotein of CH/FJWT/2018, the linear B cell epitopes in the S glycoprotein were predicted using BepiPred-2.0 server (19). As shown in Figure 3, the S1 subunit shows the most obvious differences in the distribution pattern of predicted linear B cell epitopes between CH/FJWT/2018 and GDWT-P18. Furthermore, at least 7 epitopes would be affected by amino acid variations in the S glycoprotein, five of which are located in the S1 subunit and two in the N-terminus of S2 subunit (Figures 4A–C).

## Discussion

SADS-CoV CH/FJWT/2018 was isolated from fecal and small intestinal samples of diarrheal piglets from pig farms in Fujian province, China, which represents the only virus identified outside of Guangdong province. The results in this report show that the S glycoprotein gene of CH/FJWT/2018 is distinct from other SADS-CoV isolates while the genome of those viruses shares over 99% nucleotide identities (14).

The S glycoprotein of coronaviruses mediates virus entry into host cells by binding host receptor (11, 24). Therefore, this protein determines the viral host range and tissue tropism. Several cellular proteins have been identified to be the receptors for coronaviruses. For example, angiotensin-converting enzyme 2 (ACE2) serves as the receptor for severe acute respiratory syndrome coronavirus (SARS-CoV or SARS-CoV-1), severe acute respiratory syndrome coronavirus 2 (SARS-CoV-2), and human coronavirus NL63 (HCoV-NL63) (25–27); dipeptidyl peptidase IV (DPP4) for Middle East respiratory syndrome coronavirus (MERS-CoV) (28, 29); aminopeptidase N (APN) for transmissible gastroenteritis virus (TGEV), porcine respiratory coronavirus (PRCV), and human coronavirus 229E (HCoV-229E) (30–32). For SADS-CoV, the specific receptor is still unknown and none of the known coronavirus protein receptors aforementioned are essential for the entry of SADS-CoV into the host cells (3, 33). SADS-CoV has been reported to have a broad cell tropism (33). Luo et al. further confirmed this observation as SADS-CoV isolate CN/GDWT/2017 (GenBank accession number MG557844) can efficiently replicate in 20 cell lines derived from various tissues of bat, swine, human and other animal species (34). The broad cell tropism of SADS-CoV highlights the potential of cross-species infection risk. S glycoprotein of CH/FJWT/2018 shares the lowest similarity with that of all other viral isolates (Figure 2). Moreover, 7 predicted B cell epitopes in S glycoprotein of CH/FJWT/2018 have been found to contain amino acid mutations when compared with that of other viral isolates including GDWT-P18 (Figure 3), suggesting that a significant antigenic drift may occur. Collectively, these findings emphasize that S glycoprotein gene of SADS-CoV would be an informative target either in the surveillance of SADS-CoV or in the development of preventive strategies for disease control.

The finding in this report is also reminiscent of another alphacoronavirus, feline coronavirus (FCoV) that is the causative agent for feline infectious peritonitis in wild and domestic cats (35, 36). According to the antigenic and genetic differences in the S glycoprotein, FCoV exists in two distinct types, type I and type II (37–39). Feline aminopeptidase N (fAPN) is a membrane glycoprotein with metalloproteinase activity and expressed in a variety of cells (40). It serves as a cellular receptor for the binding of S glycoprotein of FCoV type II, but not for type I, during virus entry into host cells (41–43). Therefore, the primary receptor for type I FCoV remains unknown. It has yet to be explored whether the differences in the S glycoprotein of SADS-CoVs reported in this study would affect the usage of cellular receptor or the pathogenesis of disease.

## Data Availability Statement

The datasets presented in this study can be found in online repositories. The names of the repository/repositories and accession number(s) can be found at: https://www.ncbi.nlm.nih.gov/, MH615810.1.

## Author Contributions

P-HW, Y-YG, and LX: conceptualization. P-HW, Y-YG, R-ZS, Y-QL, FG, and Y-QP: data analysis. P-HW, Y-YG, R-ZS, and LX: visualization and writing. P-HW, Y-QP, Y-YG, and LX: manuscript revision. All authors contributed to the article and approved the submitted version.

## Conflict of Interest

The authors declare that the research was conducted in the absence of any commercial or financial relationships that could be construed as a potential conflict of interest.
